# Rotator Cuff Tear Size: Could It Be Influenced by the Presence of One or More Diseases Capable of Altering the Peripheral Microcirculation?

**DOI:** 10.3390/jcm13195965

**Published:** 2024-10-07

**Authors:** Stefano Gumina, Luigi Orsina, Hyun-Seok Song, Hyungsuk Kim, Daniele Bartocci, Vittorio Candela

**Affiliations:** 1 Sapienza University of Rome, 00161 Rome, Italy; stefano.gumina@uniroma1.it (S.G.); luigi.orsina@uniroma1.it (L.O.); daniele.bartocci@uniroma1.it (D.B.); 2 Department of Orthopedic Surgery, Eunpyeong St Mary’s Hospital, School of Medicine, The Catholic University of Korea, Seoul 03312, Republic of Korea; hssongmd@hanmail.net (H.-S.S.); hskimosmd@daum.net (H.K.)

**Keywords:** rotator cuff tear, rotator cuff etiopathogenesis, rotator cuff tear size, medical conditions and rotator cuff tear, massive rotator cuff tear

## Abstract

**Background:** To date, it is not well known which systemic pathologies most frequently afflict patients with rotator cuff tear (RCT) and whether the coexistence of two or more pathologies can affect the lesion size. Therefore, we analyzed our database relative to a large group of patients who recently underwent rotator cuff repair. **Methods:** A total of 527 patients with full-thickness RCT were enrolled. For each patient, we checked the presence of at least one of diabetes, venous system diseases, cardiovascular diseases, hypercholesterolemia, blood hypertension, thyroid diseases, and a smoking habit. Patients were subdivided according to risk factors into five groups, representing those who had zero, one, two, three, and four or more risk factors, respectively. Statistical analysis was performed. **Results:** In total, 37% of our patients had no risk factors; 28% had one risk factor (arterial hypertension, smoking habit, and hypercholesterolemia were the most frequent); 23% had two risk factors (the hypertension/hypercholesterolemia association was the most frequent); and 8% suffered from three pathologies (the diabetes/arterial hypertension/hypercholesterolemia association was the most frequent). Comparing the cuff tear severity in patients without and with at least one risk factor, we observed that tear size increased in those with at least one risk factor. **Conclusions:** A total of 63% of patients with rotator cuff tears were either smokers and/or had at least one pathology capable of altering the peripheral microcirculation. Hypertension and hypercholesterolemia were the most frequent. Tear severity significantly increased with the presence of at least one risk factor.

## 1. Introduction

Degeneration and subsequent tearing of the rotator cuff (RC) have recently been attributed primarily to tissue physiological senescence and intrinsic factors, relegating extrinsic factors (such as acromion shape, slope, index, and coverage; critical shoulder angle; and acromio-clavicular deformity) to a marginal and secondary role [1,2,3,4]. Medical conditions (comorbidities) and unhealthy habits are considered the principal risk factors. Hypercholesterolemia [3,4,5], metabolic syndrome and resultant obesity [6], uncontrolled arterial hypertension [7,8], and diabetes [3,9,10] have been implicated in the pathogenesis of cuff degeneration, tearing, and re-tearing [11]. Smoking [12] and excessive alcohol consumption [13], regardless of the quality and quantity of food, are behaviors indicative of an unhealthy lifestyle. It is not uncommon for individuals to exhibit multiple intrinsic risk factors, as certain medical conditions may arise as a consequence of these habits.

The link between these medical conditions and lifestyle habits and the degeneration and tearing of the rotator cuff lies in their predisposition to peripheral microcirculatory alterations. Rothman and Parke [14], as well as Chansky and Iannotti [15], have highlighted the significant role of hypovascularity in the genesis of cuff degeneration and tearing [3,4,5].

While recent studies [3,4,5,6,7,8,9,10,11] have examined the impact of these pathologies on shoulder tendons, it remains unclear which systemic pathologies most frequently afflict patients with cuff tears and whether the coexistence of multiple pathologies affects tear size. Therefore, we conducted an analysis using our database of consecutive patients who underwent rotator cuff repair. We collected personal data, anthropometric characteristics, information on systemic diseases and lifestyle habits, cuff tear size, and details of the surgical treatment administered to each patient in order to evaluate the possible correlation between RC tear severity and comorbidities. Our hypothesis was that the presence of systemic pathologies directly affects the rotator cuff tear dimension.

## 2. Materials and Methods

### 2.1. Patient Enrollment

A consecutive series of 527 patients who underwent surgery for full-thickness rotator cuff tears from January 2019 and December 2023 were enrolled, with ages ranging from 37 to 80 years (mean age: 60.16 years).

The diagnosis was performed through physical examination and both X-ray and MRI of the affected shoulder. Among them, 37 patients (7%) reported that they began to experience shoulder problems only after direct trauma or sudden traction along the limb axis. The presence of one of the following seven risk factors was investigated for each patient: diabetes, venous system diseases, cardiovascular diseases, hypercholesterolemia, hypertension, thyroid diseases, and a smoking habit. Exclusion criteria included moderate or severe osteoarthritis of the operated or contralateral shoulder (Hamada 3 or more), prior shoulder surgery, subscapularis tendon tears, history of shoulder dislocations, concurrent or previous frozen shoulder, or inflammatory joint disease.

### 2.2. Arthroscopic Evaluation

All arthroscopic procedures were performed by one of the authors (SG) with patients under general anesthesia and interscalene block, in the beach-chair position. The Southern California Orthopedic Institute classification was used intraoperatively to classify rotator cuff tears [16]: (1) A small, complete tear, such as a puncture wound or a tear (usually <2 cm), that involves only 1 rotator cuff tendon, with no retraction of the torn ends. (2) A large, complete tear involving an entire tendon, with minimal retraction of the torn edge, usually 3 to 4 cm; and finally, (3) a massive rotator cuff tear involving 2 rotator cuff tendons, often with associated retraction and scarring of the remaining tendon ends, and frequently an irreparable L-shaped tear.

### 2.3. Groups

Patients were divided into groups based on the number of comorbidities they had, resulting in 5 groups: those with 0, 1, 2, 3, and 4 or more risk factors, respectively. Except for those with no risk factors, each group was subdivided considering all possible groupings of risk factors: 7 for 1 risk factor, 21 couples for 2 risk factors, and 35 triples for 3 risk factors. For each group, the number of small, large, and massive rotator cuff tears was counted to assess the influence of the risk factors on the severity of the lesions.

### 2.4. Statistical Analyses

The number of possible couples and triples can be derived by combinatorics. Indeed, the possible distinct couples and triples obtained from 7 risk factors are, respectively, 72=7!2!7−2!=50402⋅120=21 and 73=7!3!7−3!=50406⋅24=35 . As far as the statistical analysis is concerned, all data have been tested against the null hypothesis that the risk factors (or couples, or triples of risk factors) were evenly distributed in the population of patients, using a two-sided *p*-value on the samples. The statistically significant values were those with *p* < 0.05.

IRB: not applicable, since a standard surgical procedure has been performed and data are collected ensuring anonymity.

## 3. Results

### 3.1. Patient Characteristics

**Figure 1:**
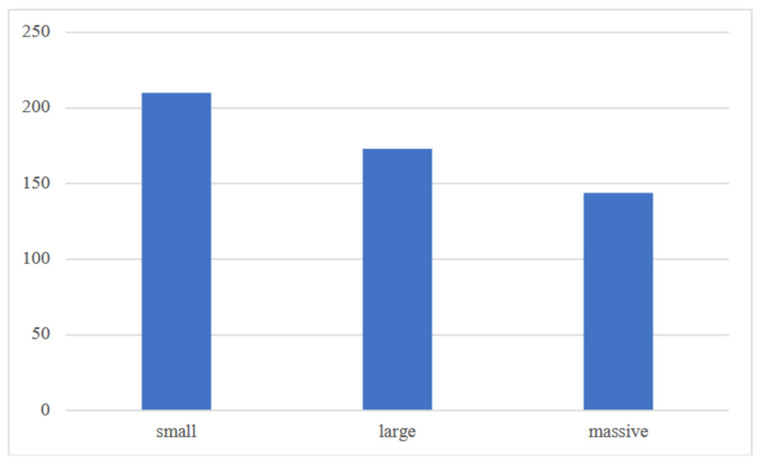
Subdivision of patients according to tear size.

Table 1 shows the subdivision of patients according to the number of risk factors, while Figure 1 shows the subdivision of patients according to tear size.

Among people with cuff tear and 1 risk factor (Table 2), which represent 27.7% of the sample, the most representative comorbidities were blood hypertension (51 patients, 35.4%), a smoking habit (29 patients, 20.1%), and hypercholesterolemia (27 patients, 18.8%).

### 3.2. Comorbidities and Cuff Tear

Cuff tear dimension in patients with blood hypertension was small in 12 cases (23.5%), large in 18 cases (35.3%), and massive in 21 cases (41.2%), respectively. A similar preponderance of large and massive tears was present in people with a smoking habit, whose lesions were small (8 patients), large (7 patients), and massive (14 patients).

### 3.3. Group Analysis

Analyzing people with exactly 2 risk factors (Table 3), which represent 23.3% of the sample, we found that the couple blood hypertension/hypercholesterolemia was present in 36 cases (29.3%), followed by blood hypertension/cardiovascular disease coupled with 18 cases (14.6%). Other couples with more than 8% of the cases were a smoking habit/hypercholesterolemia, a smoking habit/blood hypertension, diabetes/blood hypertension, and thyroid diseases/blood hypertension. As far as the gravity of the tear is concerned, in the blood hypertension/hypercholesterolemia couple, the subdivision was small with 12 (33.3%), large with 18 (50.0%), and massive with 6 (16.7%).

In our series, 43 patients (8.2%) suffered from 3 pathologies (Table 4). The most representative subgroups were diabetes/blood hypertension/hypercholesterolemia (8 cases, 18.6%), thyroid diseases/blood hypertension/hypercholesterolemia (7 cases, 16.3%), and cardiovascular diseases/blood hypertension/hypercholesterolemia (5 cases, 11.6%). In these 3 larger subgroups, which showed greater statistical significance with respect to the others, tear severity was evenly distributed among small and large/massive (9 patients with a small tear, and 5 and 6 with large and massive tears, respectively).

As far as the 23 patients with 4 or more risk factors (17 with 4 factors, 6 with 5 factors), we did not analyze the subdivisions since a large number of possible groupings (35 for 4 factors, and 21 for 5) would have shown results with little or no statistical significance. Of these patients, 8 (34.8%) had a massive tear.

Among the patients with at least one risk factor, 58.2% suffered from blood hypertension, and 40.5% from hypercholesterolemia, with the couple hypercholesterolemia/blood hypertension accounting for 39.2% of the patients with at least two risk factors.

Comparing the severity in people without and with at least one risk factor (Table 5), we observed that tear size increased when at least one risk factor was present. In fact, a small tear, according to Southern California Orthopedic Institute was observed in half of the patients who had no associated pathologies and in only a third of those with one or more risk factors. The cuff tear was large or massive in 50% and 66% of patients without and with risk factors, respectively. Table 6 shows the percentage of patients with a massive cuff tear depending on the number of risk factors.

## 4. Discussion

Thirty-seven percent of patients in our series did not have any of the systemic diseases we analyzed and that are usually considered as risk factors for cuff degeneration and tear. Therefore, in our series, responsibility for cuff tears among those who did not have a known medical condition could be mainly due to genetics [17], morphological and morphometric characteristics of the shoulder bones [18,19,20], and trauma [21], and only marginally to physiological tissue aging [22], since the average age of these patients was significantly lower than that of patients with one or more medical conditions. It is also plausible that in patients without risk factors, some disorders may have not yet manifested themselves, or have not yet been diagnosed, but are already causing damage to their target organs. On the other hand, 73% of our patients were affected by at least one pathology among those that in the literature are considered risk factors for cuff degeneration/tear. Of the pathologies that we considered, venous system diseases, thyroid diseases, and cardiovascular diseases are not among those that more often afflict patients with rotator cuff tears. While the first two pathologies have been recognized as having a marginal role in the genesis of tendon degeneration [21,23], cardiovascular diseases often represent the unfortunate consequence of diabetes, hypercholesterolemia, and hypertension; therefore, it can be deduced that patients with cardiovascular pathologies were not well represented in our series because they were recognized by anesthesiologists as unsuitable for surgery.

In our series, patients with only one risk factor frequently had blood hypertension or hypercholesterolemia or were smokers. What these risk factors have in common is tissue hypoxia, induced by alterations in the peripheral microcirculation, which appears to be a critical link [24]. Hypoxia is a crucial factor in cuff degeneration as it triggers the formation of reactive oxygen species, leading to apoptosis and a shift in cellular metabolism towards glycolysis [25,26]. These changes determine the production of intermediates such as succinate and lactic acid, which in turn enhance the secretion of interleukins (IL-6,23-1β). Hypoxia is also responsible for the downregulation of type I collagen genes and an increase in type III collagen genes in tenocytes, disrupting collagen I homeostasis [27]. Thankam and Agrawal [27], in a brilliant paper, stated that these medical conditions exacerbate these processes, thereby accelerating tendon senescence. An accumulation of reactive oxygen species causes tenocyte apoptosis through the action of mediators like cytokines, Jun N-kinase, and metalloproteinases. Over time, apoptosis results in matrix degeneration, reduced collagen synthesis, decreased mechanical tendon resistance, and, consequently, tissue degeneration and tear. These pathologies may also impact endothelial function by altering the mechanism of regulation of the bioavailability of nitric oxide [28]; the change in this mechanism is crucial because nitric oxide is the major responsible for the maintenance of vascular homeostasis in endothelial cells.

In a brilliant systematic review and meta-analysis, Giri et al. [10] stated that diabetes, hypertension, and hyperlipidemia were associated with rotator cuff; however, the possibility of bias exists for all three co-morbidities and is likely higher for hypertension. Actually, the latter bias arises because arterial hypertension is a difficult and subtle pathology to study since, as it is often asymptomatic, the diagnosis can be delayed. Furthermore, it is conceivable that in the period between the onset of symptoms and the start of pharmacological therapy, the tissues suffered an ischemic insult responsible for tendon degeneration. Furthermore, some of the most used pharmacologic therapy angiotensin-converting enzyme (ACE) inhibitors, b-blockers, and angiotensin-II receptor antagonists work more on the great vessels than on the microcirculation [10]; therefore, in patients who use these drugs, the cuff tissue continues to be in a state of hypoxia, leading to tendon degeneration. This would also explain why among those who have only one risk factor, patients with blood hypertension are those who have wider tendon tears. We have observed a similar preponderance of large and massive tears in patients with a smoking habit. Related to this, in an observational study, Baumgarten et al. [12] showed that cigarette smoking increases the risk of rotator cuff tears and their severity.

When analyzing the group of patients with two disorders, blood hypertension is almost constantly present; furthermore, the number of patients with hypertension exponentially increases when it is associated with a smoking habit (about 8%), cardiovascular diseases (about 15%), and hypercholesterolemia (about 30%), respectively. Once again, the data lead us to consider that the impairment of the peripheral microcirculation is at the basis of cuff degeneration/tear and that the association of multiple diseases also affects the tear size. In fact, in our series, patients with hypertension and hypercholesterolemia had a large or massive tear in two-thirds of the cases. Furthermore, hypertension and hypercholesterolemia were the two pathologies that we most often observed in the group of patients with three pathologies.

Finally, we observed that the severity of the tear increases with the presence of at least one risk factor.

It is completely new information that the percentage of massive tears in patients with four risk factors is more than double that observed in patients without risk factors.

The present findings underline the fundamental role of intrinsic factors and peripheral hypoperfusion in the genesis and progression of RC degeneration and tear; furthermore, it reiterates the importance of early diagnosis and treatment, especially in patients with multiple comorbidities, because this avoids the tear progression with the possible risk of high rate of re-tear or surgical failures.

Data collection regarding comorbidities is a limitation of the study: no information is present regarding the time from the diagnosis of systemic diseases nor the adhesion of the therapy; however, the effects of hypoxia have been demonstrated to be present since the beginning of the condition, as mentioned above. Furthermore, no information is present regarding alcohol consumption and obesity, which are two documented risk factors for RC degeneration, and regarding RC fatty infiltration or retraction.

## 5. Conclusions

A total of 63% of patients with rotator cuff tears were either smokers and/or had at least one pathology capable of altering the peripheral microcirculation. Of the systemic pathologies, the most frequently encountered were blood hypertension and hypercholesterolemia. Furthermore, we observed that the severity of the tear increased with the presence of at least one risk factor; the percentage of massive tears in patients with four risk factors was more than double that observed in patients without risk factors.

## Figures and Tables

**Table 1:** The studied group.

No. of Risk Factors	No. of Patients	%	Mean Age
0	194	36.8	56.8
1	144	27.3	60.1
2	123	23.3	63.5
3	43	8.2	64.1
≥4	23	4.4	63.9

**Table 2:** Distribution of patients with one risk factor (* = *p* < 0.05).

Risk Factor	No. of Patients	% of Patients	Tear Size (S, L, M)
diabetes	8	5.56 *	4, 2, 2
venous system diseases	4	2.78 *	3, 1, 0
cardiovascular diseases	10	6.94 *	5, 4, 1
hypercholesterolemia	27	18.75 *	14, 8, 5
blood hypertension	51	35.42 *	12, 18, 2
thyroid diseases	15	10.42 *	7, 5, 3
smoking habit	29	20.14 *	8, 7, 14

**Table 3:** Distribution of patients with two risk factors (* = *p* < 0.05).

Risk Factor 1	Risk Factor 2	No. of Patients	% of Patients	Tear Size (S, L, M)
diabetes	venous system diseases	0	0.00 *	0, 0, 0
diabetes	cardiovascular diseases	2	1.63 *	0, 0, 2
diabetes	hypercholesterolemia	3	2.44 *	2, 0, 1
diabetes	blood hypertension	11	8.94 *	4, 4, 3
diabetes	thyroid diseases	0	0.00 *	0, 0, 0
diabetes	smoking habit	1	0.81 *	1, 0, 0
venous system disease	cardiovascular diseases	1	0.81 *	0, 1, 0
venous system diseases	hypercholesterolemia	2	1.63 *	0, 2, 0
venous system diseases	blood hypertension	3	2.44 *	1, 1, 1
venous system diseases	thyroid diseases	1	0.81 *	0, 1, 0
venous system diseases	smoking habit	0	0.00 *	0, 0, 0
cardiovascular diseases	hypercholesterolemia	3	2.44 *	1, 0, 2
cardiovascular diseases	blood hypertension	18	14.63 *	6, 6, 6
cardiovascular diseases	thyroid diseases	2	1.63 *	1, 1, 0
cardiovascular diseases	smoking habit	1	0.81 *	1, 0, 0
hypercholesterolemia	blood hypertension	36	29.27 *	12, 18, 6
hypercholesterolemia	thyroid diseases	5	4.07	0, 4, 1
hypercholesterolemia	smoking habit	10	8.13 *	3, 2, 5
blood hypertension	thyroid diseases	11	8.94 *	2, 7, 2
blood hypertension	smoking habit	10	8.13 *	2, 3, 5
thyroid diseases	smoking habit	3	2.44 *	2, 1, 0

**Table 4:** Distribution of patients with three risk factors (* = *p* < 0.05).

Risk Factor 1	Risk Factor 2	Risk Factor 3	No. of Patients	% of Patients	Tear Size (S, L, M)
diabetes	venous system diseases	cardiovascular diseases	0	0.00	0, 0, 0
diabetes	venous system diseases	hypercholesterolemia	1	2.33	0, 0, 1
diabetes	venous system diseases	blood hypertension	0	0.00	0, 0, 0
diabetes	venous system diseases	thyroid diseases	1	2.33	1, 0, 0
diabetes	venous system diseases	smoking habit	0	0.00	0, 0, 0
diabetes	cardiovascular diseases	hypercholesterolemia	0	0.00	0, 0, 0
diabetes	cardiovascular diseases	blood hypertension	3	6.98 *	2, 1, 0
diabetes	cardiovascular diseases	thyroid diseases	0	0.00	0, 0, 0
diabetes	cardiovascular diseases	smoking habit	0	0.00	0, 0, 0
diabetes	hypercholesterolemia	blood hypertension	8	18.60 *	4, 2, 2
diabetes	hypercholesterolemia	thyroid diseases	1	2.33	1, 0, 0
diabetes	hypercholesterolemia	smoking habit	2	4.65 *	1, 0, 1
diabetes	blood hypertension	thyroid diseases	0	0.00	0, 0, 0
diabetes	blood hypertension	smoking habit	2	4.65 *	1, 1, 0
diabetes	thyroid diseases	smoking habit	0	0.00	0, 0, 0
venous system diseases	cardiovascular diseases	hypercholesterolemia	2	4.65 *	0, 2, 0
venous system diseases	cardiovascular diseases	blood hypertension	2	4.65 *	1, 0, 1
venous system diseases	cardiovascular diseases	thyroid diseases	0	0.00	0, 0, 0
venous system diseases	cardiovascular diseases	smoking habit	0	0.00	0, 0, 0
venous system diseases	hypercholesterolemia	blood hypertension	1	2.33	0, 1, 0
venous system diseases	hypercholesterolemia	thyroid diseases	1	2.33	1, 0, 0
venous system diseases	hypercholesterolemia	smoking habit	0	0.00	0, 0, 0
venous system diseases	blood hypertension	thyroid diseases	1	2.33	0, 1, 0
venous system diseases	blood hypertension	smoking habit	2	4.65 *	0, 1, 1
venous system diseases	thyroid diseases	smoking habit	0	0.00	0, 0, 0
cardiovascular diseases	hypercholesterolemia	blood hypertension	5	11.63 *	2, 0, 3
cardiovascular diseases	hypercholesterolemia	thyroid diseases	0	0.00	0, 0, 0
cardiovascular diseases	hypercholesterolemia	smoking habit	0	0.00	0, 0, 0
cardiovascular diseases	blood hypertension	thyroid diseases	0	0.00	0, 0, 0
cardiovascular diseases	blood hypertension	smoking habit	2	4.65 *	0, 0, 2
cardiovascular diseases	thyroid diseases	smoking habit	0	0.00	0, 0, 0
hypercholesterolemia	blood hypertension	thyroid diseases	7	16.28 *	3, 3, 1
hypercholesterolemia	blood hypertension	smoking habit	2	4.65 *	0, 0, 2
hypercholesterolemia	thyroid diseases	smoking habit	0	0.00	0, 0, 0
blood hypertension	thyroid diseases	smoking habit	0	0.00	0, 0, 0

**Table 5:** Distribution of patients according to tear size and presence of risk factors.

	Tear Size		
No. of Risk Factors	Small (%)	Large (%)	Massive (%)
0	97 (50.0%)	55 (28.4%)	42 (21.7%)
≥1	113 (33.9%)	118 (35.4%)	102 (30.6%)

**Table 6:** Distribution of massive cuff tears among patients depending on the number of risk factors.

Number of Risk Factors	% of Massive Tears
0	21.6%
1	31.9%
2	27.6%
3	32.6%
4	47.1%

## Data Availability

The data presented in this study are available on request from the corresponding author.
